# Lobe-Specific Calcium Binding in Calmodulin Regulates Endothelial Nitric Oxide Synthase Activation

**DOI:** 10.1371/journal.pone.0039851

**Published:** 2012-06-29

**Authors:** Pei-Rung Wu, Cheng-Chin Kuo, Shaw-Fang Yet, Jun-Yang Liou, Kenneth K. Wu, Pei-Feng Chen

**Affiliations:** Institute of Cellular and System Medicine, National Health Research Institutes, Zhunan, Miaoli County, Taiwan; Griffith University, Australia

## Abstract

**Background:**

Human endothelial nitric oxide synthase (eNOS) requires calcium-bound calmodulin (CaM) for electron transfer but the detailed mechanism remains unclear.

**Methodology/Principal Findings:**

Using a series of CaM mutants with E to Q substitution at the four calcium-binding sites, we found that single mutation at any calcium-binding site (B1Q, B2Q, B3Q and B4Q) resulted in ∼2–3 fold increase in the CaM concentration necessary for half-maximal activation (EC50) of citrulline formation, indicating that each calcium-binding site of CaM contributed to the association between CaM and eNOS. Citrulline formation and cytochrome c reduction assays revealed that in comparison with nNOS or iNOS, eNOS was less stringent in the requirement of calcium binding to each of four calcium-binding sites. However, lobe-specific disruption with double mutations in calcium-binding sites either at N- (B12Q) or at C-terminal (B34Q) lobes greatly diminished both eNOS oxygenase and reductase activities. Gel mobility shift assay and flavin fluorescence measurement indicated that N- and C-lobes of CaM played distinct roles in regulating eNOS catalysis; the C-terminal EF-hands in its calcium-bound form was responsible for the binding of canonical CaM-binding domain, while N-terminal EF-hands in its calcium-bound form controlled the movement of FMN domain. Limited proteolysis studies further demonstrated that B12Q and B34Q induced different conformational change in eNOS.

**Conclusions:**

Our results clearly demonstrate that CaM controls eNOS electron transfer primarily through its lobe-specific calcium binding.

## Introduction

Human endothelial nitric oxide synthase (eNOS) catalyzes the synthesis of nitric oxide (•NO), which is a key regulator of cardiovascular homeostasis [1], [2]. Like neuronal NOS (nNOS) [3], [4] and macrophage inducible NOS (iNOS) [5], [6], eNOS [7] is a homodimer; each monomer contains a C-terminal reductase domain, and an N-terminal oxygenase domain [8]–[10]. The reductase domain binds FAD and FMN cofactors, and consists of NADPH binding site, while the oxygenase domain binds protoporphrin IX heme, tetrahydrobiopterin (H_4_B), and contains the site for L-arginine binding. These two domains are connected by a canonical CaM-binding sequence. Binding of calcium/CaM to this site facilitates intra-domain electron transfer between FAD and FMN as well as inter-domain electron transfer from the flavin of one subunit to the heme of the other subunit [11]–[13]. Although the mechanism by which CaM regulates NOS activation has been under intensive investigation, much remains unresolved. Our recent work on eNOS has demonstrated that in addition to the putative CaM binding domain (residues 491–510, hereafter referred to simply as CBD) [14], two other regions, *i.e.* residues 174–193 of the heme-binding site and residues 729–757 located at the hinge region connecting FAD and FMN subdomains, are possibily involved in CaM/calcium-regulated eNOS activation and deactivation [15]. Thus, the mechanisms underlying CaM/calcium-dependent eNOS catalysis are more complicated than previously thought.

CaM is a ubiquitous calcium-binding protein, structurally resembling a dumbbell. It contains N- and C-terminal lobes joined by a flexible helical linker [16]. Each lobe comprises two E–F hands with two calcium-binding sites. The calcium-binding sites in the C-terminal lobe (sites 3 and 4) have a higher affinity for calcium than those in the N-terminal lobe (sites 1 and 2) [17]. Calcium binding triggers conformational changes of CaM, bringing the two lobes together, forming a hydrophobic interface to interact with its target proteins [18].

CaM regulates all three NOS isoforms. iNOS binds CaM tightly at low calcium level and is catalytically active at basal cellular calcium concentrations [19], while eNOS and nNOS bind CaM reversibly in a calcium concentration-dependent manner and require high calcium concentrations for catalysis [7], [20]. Previous reports using CaM mutants with different calcium-binding site mutations suggest differential roles of the four calcium-binding sites for nNOS and iNOS in regulating CaM binding and electron transfer [21]–[23]. The role that each calcium- binding site plays in regulating CaM binding to eNOS and eNOS catalytic activity is unclear. As eNOS is attached to plasma membrane through myristoylation and palmitaylation, calcium-CaM dependent electron transfer and catalysis are likely to be influenced by diverse signaling pathways making it more complicated than iNOS or nNOS [24]. Furthermore, since the calcium-binding sites of CaM can differentially regulate target protein function, and a large fraction of free CaM is not fully occupied with four calcium ions in fluctuating intracellular calcium concentration [25], it is essential to clarify the role of each calcium-binding site of CaM in eNOS activity.

**Figure 1:**
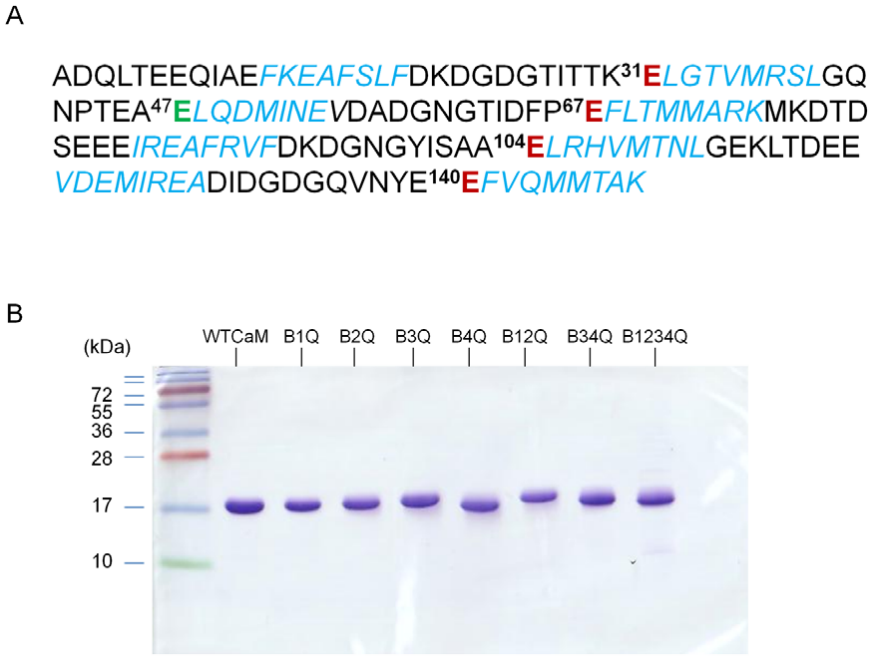
CaM mutant constructs. A: The sequence of human CaM is shown, using single-letter amino acid residue codes. The mutated amino acid residue in each calcium-binding site is marked in red. Helical regions are shown in Italics and blue color. B: The electrophoretic mobility of wild-type and mutant CaM proteins was analyzed by SDS-PAGE. A 5-μg sample of each CaM construct was loaded in a 15% SDS-PAGE which was run with a standard Laemmli SDS-PAGE buffer in the presence of 1 mM EGTA. Molecular masses of protein standards are shown on the left in kilodaltons (kDa).

**Table 1:** The primers used for mutations and the residues changed for each mutant.

Mutants	Primers	Residue changed
B1Q	5′-CACCACCAAG**CAA**TTGGGGACAGTGAT-3′	E_31_–Q_31_
	5′-ATCACTGTCCCCAA**TTG**CTTGGTGGTG-3′	
B2Q	5′-CTTCCCG**CAG**TTCTTAACCATGATG-3′	E_67_–Q_67_
	5′-CATCATGGTTAAGAA**CTG**CGGGAAG-3	
B3Q	5′-CTACATCAGCGCTGCG**CAG**CTGCGT-3′	E_104_–Q_104_
	5′-ACGCAG**CTG**CGC AGCGCTGATGTAG-3′	
B4Q	5′-GGCCAGGTTAACTATGAA**CAG**TTTGTA-3′	E_140_–Q_140_
	5′-TACAAA**CTG**TTCATAGTTAACCTGGCC-3′	
B12Q		E_31_–Q_31_
		E_67_–Q_67_
B34Q		E_104_–Q_104_
		E_140_–Q_140_
B1234Q		E_31_–Q_31_
		E_67_–Q_67_
		E_104_–Q_104_
		E_140_–Q_140_

Electron flow in NOS isoforms is controlled by CaM/calcium binding. In the absence of CaM, the FMN domain is in a shielded state (input) which is locked by NADPH binding [26] and stabilized by the relative spatial arrangements of autoinhibitory elements and C-terminal tail. In this state, the FMN domain is in close contact with the FAD/NADPH binding domain [27], and electron transfer is limited to FAD to FMN and not to other electron acceptors. CaM/calcium binding elicits a swing of FMN domain toward a deshielded (output) state, which facilitates electron transfer to heme/H_4_B or cytochrome c [28]–[31]. The N- and C-terminal lobes of CaM bind calcium ions with different affinities and lobe-specific functions of CaM are frequently seen in the regulation of its target proteins [32], [33]. We hypothesized that the shift of FMN subdomain from input to output states for electron transfer depends on lobe-specific calcium/CaM binding to eNOS. To test this hypothesis, we mutated a single calcium-binding site (designated B_1Q_, B_2Q_, B_3Q_ and B_4Q_, respectively), both binding sites at N-lobe (B_12Q_) or C-lobe (B_34Q_) or all four calcium-binding sites (B_1234Q_), and assessed how these mutations impact CaM binding to CaM binding domain and CaM-mediated eNOS conformational change, and catalytic activities.

## Results

### CaM mutants in eNOS activation

CaM mutants carrying various E to Q substitutions at calcium-binding sites were generated based on the sequence of human CaM shown in Figure 1A. Primers used to generate CaM mutants and the designated names for each mutant were listed in Table 1. Purified CaM mutants were at least 95% pure with an estimated molecular mass of ∼17 kDa based on SDS-PAGE analysis, which was run in a standard SDS-PAGE buffer containing 1 mM EGTA. We generally obtained about 16 to 100 mg purified proteins from a liter of culture medium. The electrophoretic mobility of most mutants was not obviously different from that of the wild-type CaM, with the exception of B_4Q_ mutant which had slightly higher mobility (Figure 1B).

**Figure 2:**
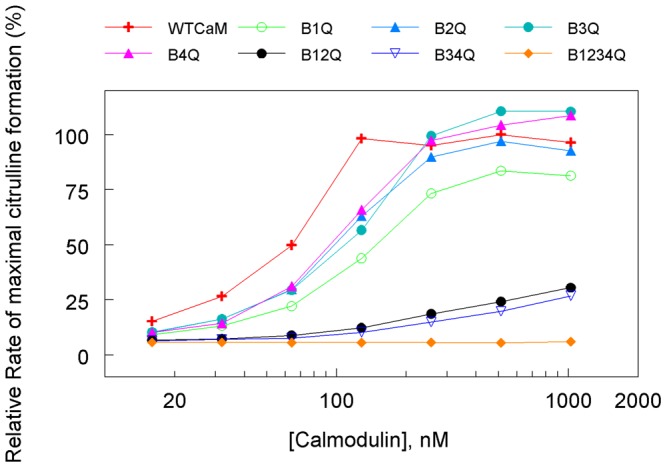
Citrulline formation as a function of CaM concentrations. Citrulline forming activity was determined in a reaction mixture containing 25 mM Tris, pH 7.5, 100 nM eNOS, 3H-L-arginine (**1**μCi), L-arginine (20 μM), NADPH (100*μ*M), H_4_B (10*μ*M), and CaCl_2_ (300 μM), 0.2 mM EDTA, 100 mM NaCl, 0.1 mM DTT, 10% glycerol and the indicated concentration of each CaM construct as described under Experimental Procedures. Data are mean of duplicate measurements with <5% variation. Each experiment was repeated three times.

The steady-state citrulline formation was used to determine electron flux from NADPH to the heme of oxygenase domain. Effects of wild-type and mutant CaMs on citrulline formation was evaluated by adding increasing concentrations of CaM to the assay mixture. Concentration response curves for wild-type CaM and each mutant are shown in Figure 2. Compared to wild-type CaM, B_1234Q_ showed no activation at all while B_12Q_ and B_34Q_ greatly diminished ability to activate citrulline formation through their wide-ranging concentrations. The response curves of the four single-site mutants (B_1Q_, B_2Q_, B_3Q_, and B_4Q_) resembled the wild-type response curve. Quantitative analysis from multiple experiments revealed that the EC_50_ (concentration required for half maximal citrulline formation) for the single-site mutants is about 2–3 fold higher than that of wild-type (Table 2). These data suggest that each of the four calcium-binding sites supports the association between CaM and eNOS (Table 2).

**Table 2:** The EC_50_ for each CaM construct in activation of eNOS citrulline formation.

Mutants	EC_50_ (nM)
WTCaM	75±10
B1Q	161±14
B2Q	125±12
B3Q	170±16
B4Q	148±8

The *bar values* represent the means ± standard deviation. The mean values were obtained from three independently prepared batches of proteins.

**Figure 3:**
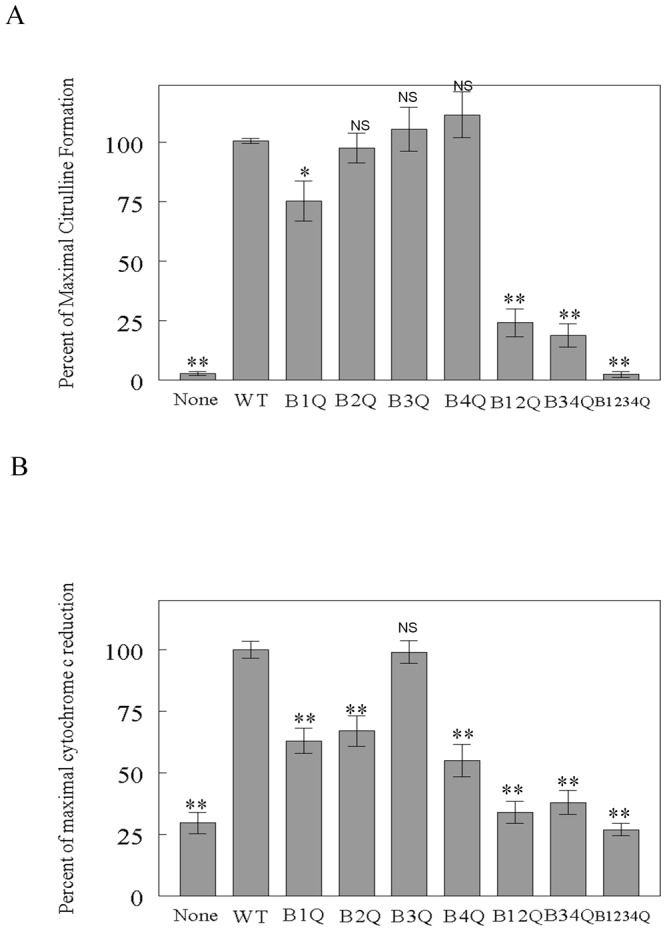
Ability of various CaM mutants to activate eNOS enzymes. A: Citrulline formation activity. B: *Cytochrome c* reduction activity. Both citrulline formation and *cytochrome c* reduction were measured in the presence of saturating concentration of each CaM construct (0.5 μM). NOS activities were expressed as percentage of the respective maximal NOS activity (100%) which was obtained with the eNOS bound to wild-type CaM. Under these conditions, the activities for eNOS bound to wild-type CaM were ∼14 min^−1^ for citrulline formation, and ∼286 min^−1^ for *cytochrome c* reduction. Data are presented as means±standard deviation. * denotes *p<*0.05, ** <0.01, NS, not significant difference as compared to wild-type CaM. Each experiment was performed in triplicate and repeated three times.

As CaM concentrations increased, some mutants exhibited citrulline forming activity even higher than wild-type CaM. We compared the citrulline formation in the presence of saturating concentrations for each CaM construct (0.5 μM), and activity obtained with eNOS bound to wild-type CaM was set to 100%. As shown in Figure 3A, the wild-type CaM increased citrulline formation ∼50-fold above that observed in the absence of CaM. Maximal citrulline formation with B_1Q_ was reduced by 25% and B_2Q_ was not significantly different from the wild-type (Figure 3A), while maximal citrulline formation of B_3Q_ and B_4Q_ was slightly higher than that of the wild-type. Of the two double mutants, the extent of maximal activity with B_12Q_ and B_34Q_ was reduced to 24% and 18% of the wild-type, respectively. The activity with B_1234Q_ was not increased and remained at the level without CaM (Figure 3A). The result indicates that except for B_1Q_, single mutation at calcium-binding site of CaM has little effect on eNOS citrulline forming activity while disruption in two calcium-binding sites either at N- (B_12Q_) or at C-terminal (B_34Q_) lobe greatly impacts eNOS oxygenase activity.

We next evaluated the effect of CaM mutants on eNOS reductase activity. *Cytochrome c* accepts electrons exclusively from the FMN domain and has been used to measure electron flow within the reductase domain. Binding of wild-type CaM to eNOS induced a ∼4-fold increase in *cytochrome c* reduction. B_3Q_ mutant increased *cytochrome c* reduction to a level comparable to wild-type CaM (Figure 3B). On the other hand, *cytochrome c* reduction induced by B_1Q_, B_2Q_ and B_4Q_ was 63%, 67% and 55% of the wild-type level, respectively. The multiple mutants, *i.e.* B_12Q_, B_34Q_ and B_1234Q_ exhibited slight or no increase in *cytochrome c* reductase activity. These results demonstrate that with the exception of B_3Q_, single mutation at calcium-binding sites in CaM affects the reductase activity greater than the oxygenase activity in eNOS. Furthermore, full calcium bindings at N- and C-terminal lobes are essential for electron transfer through the eNOS reductase domain (Figure 3B).

### Binding of CaM mutants to eNOS CaM binding domain (CBD) Peptide

**Figure 4:**
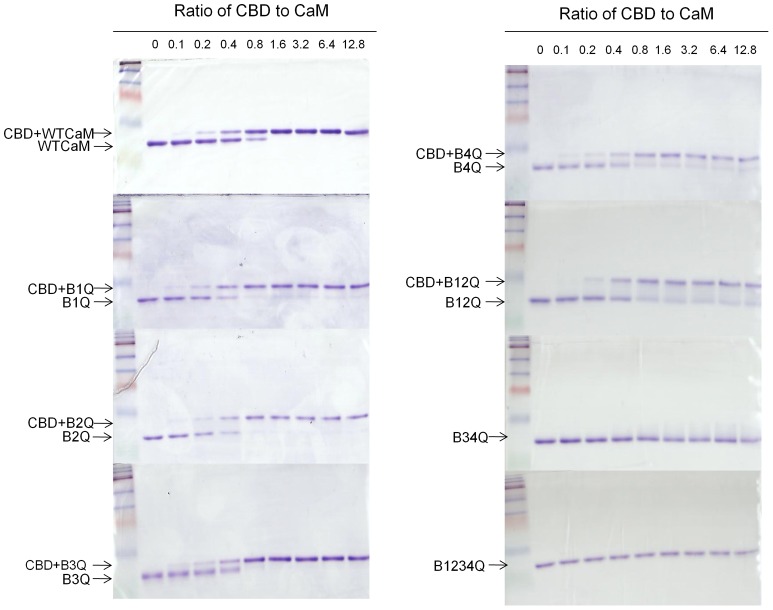
Interaction of CaM-binding domain with each CaM construct. The canonical CaM-binding domain of human eNOS (CBD, residues 491–510) was incubated with CaM constructs (200 pmol) by increasing peptide:CaM molar ratios at room temperature for 1 h before electrophoresis in the presence of 100 μM of calcium. The samples were analyzed on 18% non-denaturing gels and visualized with Coomassie Blue R-250. The first lane in each gel contains CaM only, *i.e.* CBD/CaM ratio is 0. The rest CBD/CaM ratios were indicated. CBD-CaM complexes and free CaM are denoted.

The ability of various CaM mutants to bind CBD was evaluated. Each CaM mutant was incubated with CBD in the presence of 100 μM calcium at several different peptide:CaM ratios. The CBD-CaM complexes and free CaM were examined by nonreducing, nondenaturing electrophoresis. Free peptides were not detected because they are positively charged at neutral pH and therefore will have an upward mobility toward cathode and do not enter the gel [34], [35]. In the presence of EGTA, no mobility shift bands were detected for wild-type or any CaM mutant (data not shown). Peptide-CaM complexes can be seen above free CaM band in the presence of 100 μM calcium. The extent of CaM-peptide interaction is assessed by measuring attenuation of free CaM bands and the shift in the mobility of the CaM protein with increasing peptide concentration. As shown in Figure 4, B_1Q_, B_2Q_ and B_3Q_ bound CBD with an affinity similar to the wild-type CaM. B_4Q_ and B_12Q_ bound CBD but the binding was not complete. B_34Q_ and B_1234Q_ did not bind CBD. These results suggest that the canonical CaM-binding site on eNOS interacts with the calcium-bound C-lobe in CaM.

### Distinct roles of CaM N- and C-Terminal lobes on eNOS Flavin Fluorescence

**Figure 5:**
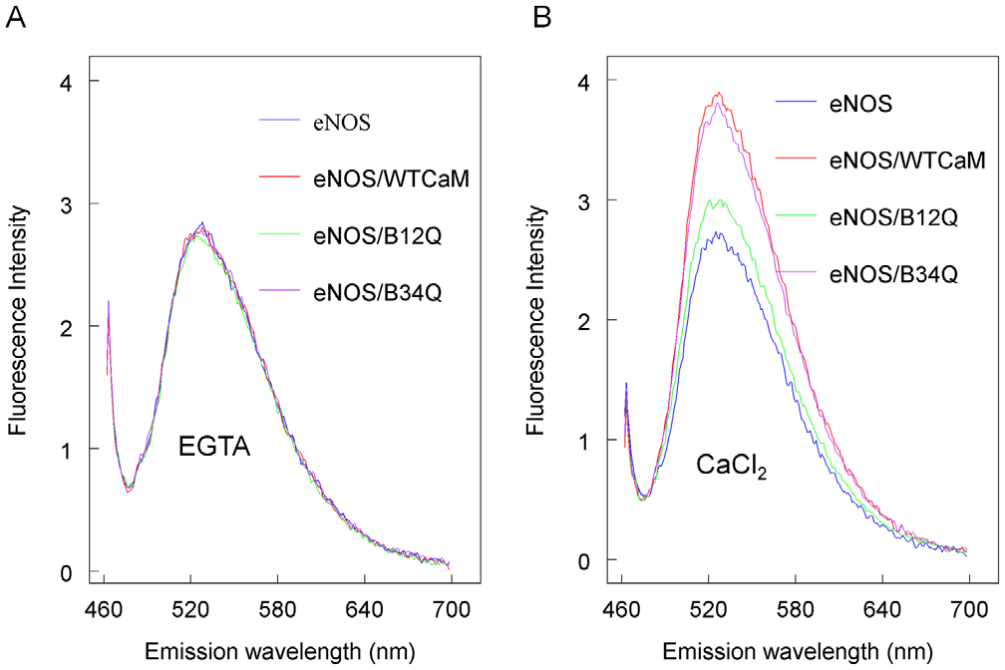
Flavin fluorescence of eNOS treated with wild-type and CaM mutants. A: Fluorescence emission spectra of eNOS were obtained in a solution containing 25 mM Tris, pH 7.5, 100 mM NaCl, 0.1 mM DTT, ∼6 μM eNOS and ∼12μM of wild-type CaM, B_12Q_ or B_34Q_ in the presence of 2 mM EGTA. B: The change of fluorescence intensity for wild-type CaM, B_12Q_ or B_34Q_ was recorded upon addition of 2.5 mM CaCl_2_ to the above mixtures. Excitation wavelength was set at 455 nm. The fluorescence data were corrected by subtracting CaM and buffer effects. The eNOS alone and eNOS-CaM complex are indicated in each plot.

Since the N- and C-terminal lobes of CaM can function as an independent domain [33], [36], we determined whether a conversion of the FMN subdomain from shielded to deshielded state depends on calcium-bound N- and C-terminal lobes of CaM. As the intensity of flavin fluorescence is proportional to the degree of FMN deshielding [37], we monitored the change in flavin fluorescence of eNOS induced by wild-type CaM, B_12Q_ or B_34Q_ in the presence or absence of calcium. As shown in Figure 5A, wild-type and mutant CaMs were unable to increase flavin fluorescence of eNOS in the presence of 2 mM EGTA. Upon addition of calcium, B_34Q_ increased flavin fluorescence intensity to the same level as the wild-type CaM. In contrast, B_12Q_ increased much less fluorescence intensity compared with wild-type CaM or B_34Q_, indicating that the N-terminal EF-hands in its calcium-bound form are responsible for the input/output swing of FMN domain (Figure 5B).

### Trypsinolysis of eNOS in the presence of wild-type CaM, B12Q or B34Q

As the results shown above have provided evidence that CaM N- and C-terminal lobes in its calcium-bound form play distinct roles in interacting with CaM binding domain and in the shift of input/output state of FMN domain, we speculated that binding of these two lobes might induce different conformational changes in eNOS. We used limited trypsin digestion to detect the eNOS conformation changes induced by wild-type vs. B_12Q_ or B_34Q_. Limited trypsinolysis of eNOS was carried out in the presence of high or low calcium concentrations.

**Figure 6:**
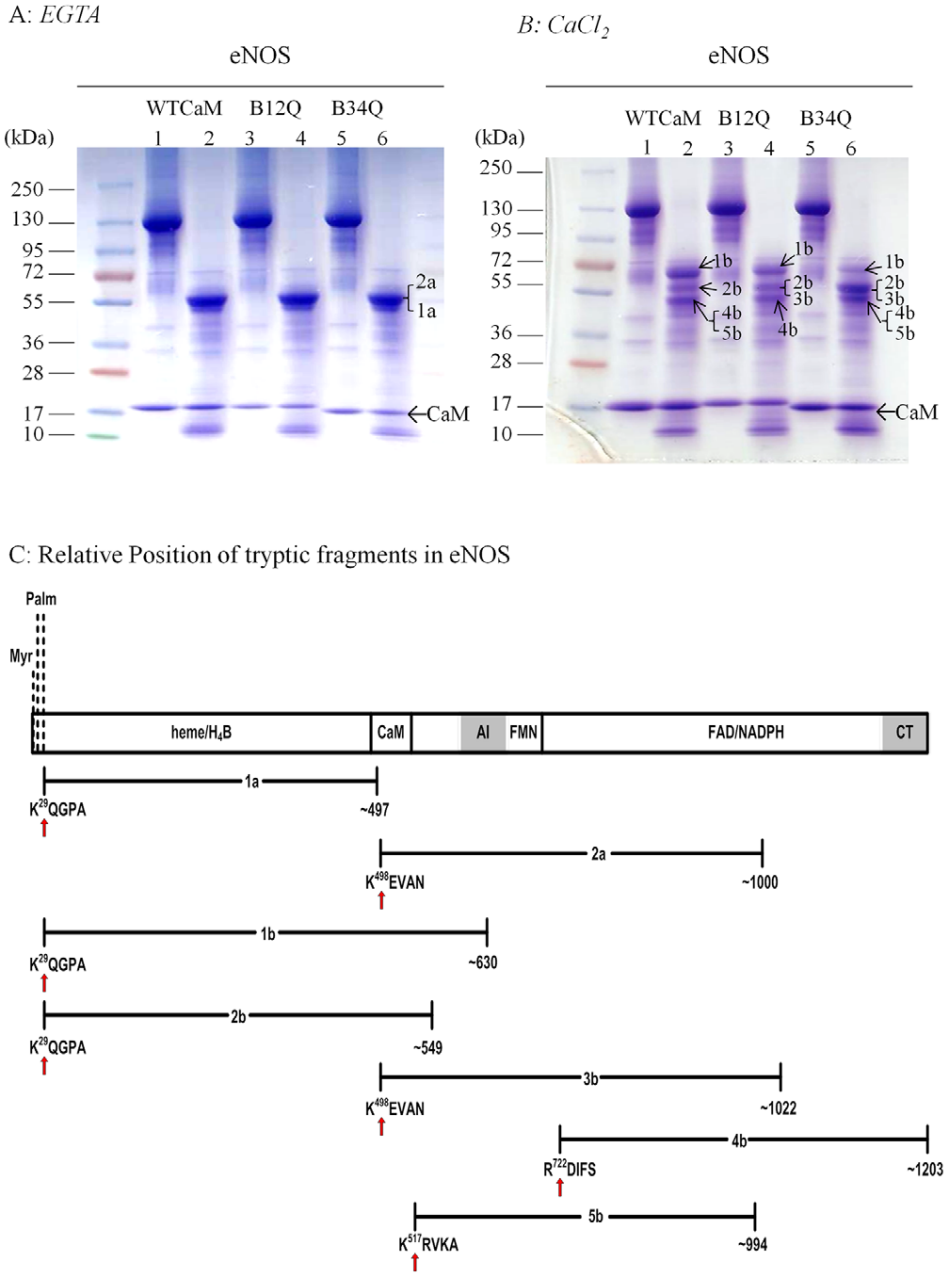
Limited trypsinolysis of eNOS in the presence of wild-type CaM, B_12Q_ or B_34Q_. A: Trypsinolysis of eNOS (∼5μM) was performed in the presence of 10 μM of wild-type CaM, B_12Q_ or B_34Q_ at 22°C for 15 min with 1 mM EGTA. B: Trypsinolysis was carried out in the presence of 100 μM CaCl_2_. Lane 1: undigested eNOS with wild-type CaM; lane 2: trypsinized eNOS with wild-type CaM; lane 3: undigested eNOS with B_12Q_; lane 4: trypsinized eNOS with B_12Q_; lane 5: undigested eNOS with B_34Q_; lane 6: trypsinized eNOS with B_34Q_. Molecular masses of marker proteins are shown on the *left* in kilodaltons (kDa). Following SDS-PAGE, the tryptic samples were transferred to PVDF membranes using CAPS buffer for 3 h at 50 V and 4°C. The bands excised for N-terminal sequencing are indicated. C: Relative position of N-terminal sequences of the indicated tryptic fragments in the schematic eNOS structure. The C-terminal termination sites are unknown and the residue at C-terminal end in each tryptic fragment was approximately estimated from the apparent molecular mass. Red arrow depicts the tryptic cleavage site. The blocks indicate binding sites for heme, H_4_B, CaM, FMN, FAD and NADPH. AI and CT refer to autoinhibitory loop and C-terminal tail, respectively. The myristoylation site (Myr.) and palmitoylation sites (Palm.) are denoted.

In the absence of calcium, trypsinolysis of eNOS incubated with wild-type, B_12Q_ or B_34Q_ produced an identical fragment profile with one major band having apparent molecular mass of ∼ 56 kDa. N-terminal sequencing revealed that this band was composed of two portions of N-terminal sequences of K^29^QGPA (fragment-1a in Figure 6A) and K^498^EVAN (fragment-2a in Figure 6A), indicating two cleavage sites: (1) between Lys28 and Gln29 and (2) between Lys497 and Glu498. Lys28 is close to the palmitoylation site, and Lys497 is within the canonical CaM-binding domain of eNOS (Figure 6C), reflecting that without CaM protection, CaM-binding domain of eNOS is sensitive to trypsin digestion.

In the presence of calcium, trypsin digestion of eNOS with wild-type CaM yielded three notable bands ∼67 kDa (fragment-1b), ∼58 kDa (fragment-2b) and ∼52 kDa(fragment-4b & 5b) (Figure 6B). N-terminal sequencing of ∼67 kDa band revealed the cleavage site at K^29^QGPA and judged from the apparent size, the C-terminal tryptic site is probably around Arg630 which is situated in the autoinhibitory (AI) region (Figure 6C, fragment-1b), consistent with the previous report that calcium/CaM binding induces eNOS conformational change resulting in the exposure of the autoinhibitory elements [38]. N-terminal sequencing of the ∼58-kDa band showed the cleavage site at K^29^QGPA and C-terminal tryptic residue at approximately 549, based on size estimate (fragment-2b in Figure 6C). N-terminal sequencing of ∼52 kDa band revealed two N-terminal sequences: (1) fragment-4b with N-terminal sequence of R^722^DIFS and an estimated C-terminal end at residue 1203, and (2) fragment-5b with N-terminal sequence of K^517^RVKA and an estimated C-terminal end at residue 994 (Figure 6C).

Trypsin digestion of eNOS with B_12Q_ produced a similar fragment profile as that with wild-type CaM, except that a portion of N-terminal sequence of fragment-3b was not detected in wild-type CaM and fragment 5b was not detected in B_12Q_ (Figure 6B). The N-terminal sequence of fragment-3b is K^498^EVAN that is within canonical CaM-binding domain at eNOS (Figure 6C). By contrast, the fragment profile of eNOS trypsinolysis with B_34Q_ revealed an attenuated fragment-1b and enhanced fragment-2b and 3b as well as fragments 4b and 5b. The trypsinolysis data provide important insights into the influence of CaM on eNOS conformation: (1) Cleavage of eNOS at Lys28 was observed either in the presence or absence of CaM, indicating that the sequence at this residue near the palmitoylation site is in an exposed area. The eNOS is dually acylated by *N*-myristoylation at Gly2 and by thiopalmitoylations at Cys15 and Cys26 [39], [40]. As palmitoylation is reversible, whether existence of regulated cycles of palmitoylation and depalmitoylation controls eNOS degradation remains further investigation; (2) N-terminal sequence of K^498^EVAN (fragment-3b) was detected with B_12Q_ and B_34Q_ but not with wild-type CaM; the intensity of fragment-3b in B_12Q_ was weaker than in B_34Q_. This is consistent with the gel mobility shift assay, in which B_12Q_ binds CaM binding domain but not as well as wild-type CaM; B_34Q_ shows no binding at all. The result further confirms that C-terminal lobe in its calcium-bound form can protect eNOS CaM binding domain from tryptic digest; (3) binding of eNOS by wild-type, B_12Q_ or B_34Q_ similarly resulted in the exposure of R^722^DIFS to trypsin digestion. This sequence is situated at the hinge region between FMN and FAD domains (Figure 6C). Our previous finding has suggested that this region may interact with CaM and is involved in eNOS catalysis [15]. That binding of wild-type or CaM mutants impaired in calcium-binding at C- or N-lobe caused a similar exposure of the hinge region, suggests highly flexible nature of this region, which is likely to serve as a pivot for the motion of FMN domain [41]; (4) Exposure of K^517^RVKA (fragment-5b) with wild-type and B_34Q_ binding but not B_12Q_ binding suggests that binding of CaM N-terminal lobe in calcium-bound form exposes Arg517 for trypsin digestion. eNOS Arg517 is analogous to iNOS Arg536 and nNOS Arg757 which are reported to form both salt bridge and hydrogen bonding interaction with CaM Glu47 and several backbone oxygens in NOS FMN subdomain [42], and are considered to be crucial for control of FMN subdomain interactions with its redox partners [43].

## Discussion

The results indicate that each calcium-binding site on CaM plays a part in mediating CaM-induced eNOS electron transfer and catalytic activity. Single mutation of each binding site results in an increased EC50 by ∼2–3 fold over the wild-type without a substantial effect on maximal citrulline formation. However, double mutation of binding sites at the C-lobe or N-lobe of CaM drastically reduces maximal citrulline formation and *cytochrome c* reduction, indicating requirements of lobe-specific calcium binding for eNOS reductase and oxygenase activities. Gel mobility shift assay, flavin fluorescence measurements and limited trypsinolysis demonstrate that the N- and C-terminal lobes play a distinct role in eNOS activation, *i.e.* N-lobe in its calcium-bound form controls FMN subdomain motion, while C-lobe in its calcium-bound form binds CaM binding domain region in eNOS.

**Figure 7:**
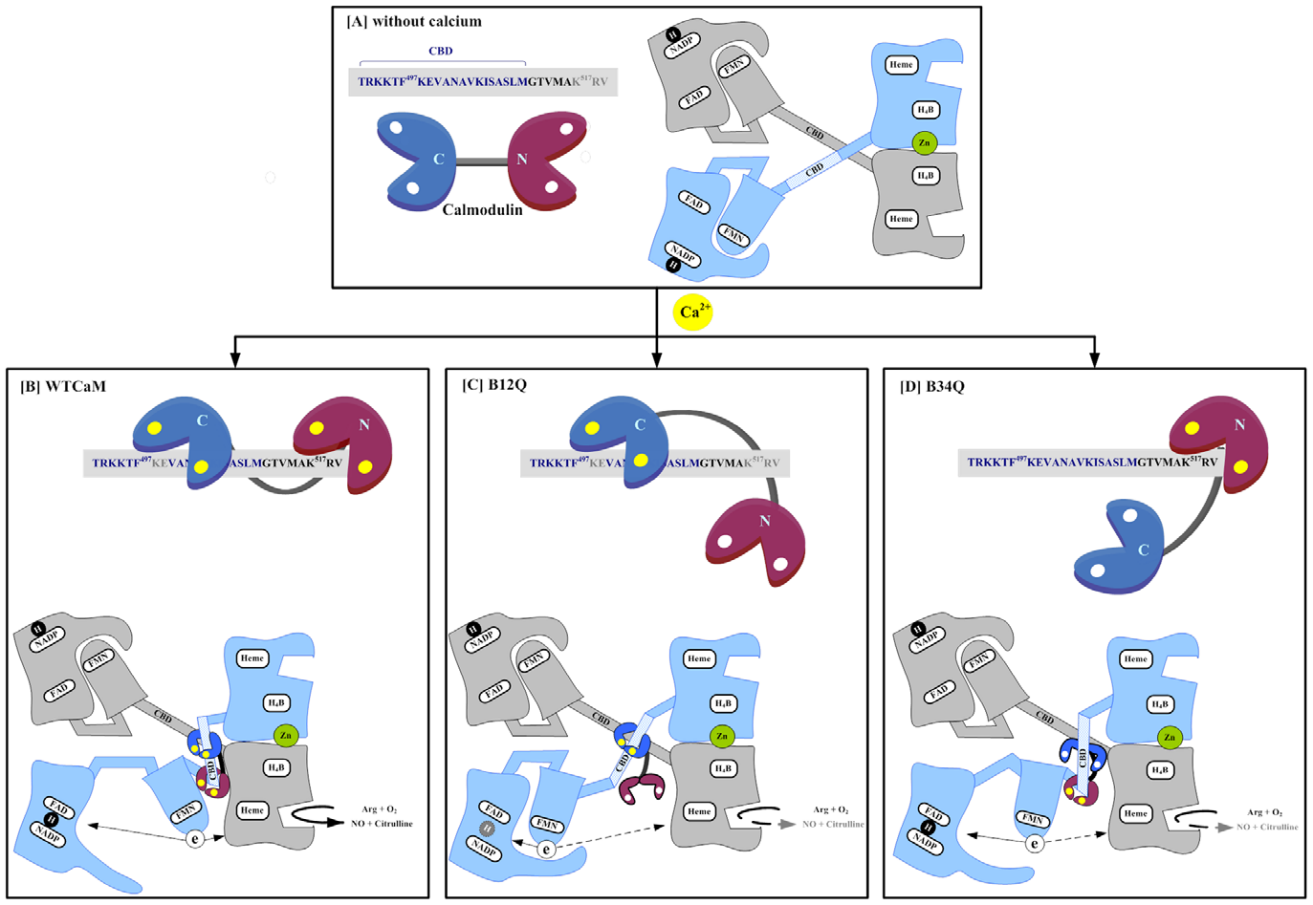
Schematic illustration of differential regulation of eNOS electron transfer by N- and C-terminal lobes of CaM. Dimeric eNOS model is derived from Daff [44]. The *gray colored* domains represent one subunit, and the *sky-blue colored* domains represent the other. The blocks indicate binding sites for heme, H_4_B, zinc, CaM, FMN, FAD and NADPH. The canonical CaM-binding domain (residues 491–510) between the FMN domain and the heme domain is denoted as CBD. CaM shown as two *lobed structures* N and C, joined by a flexible linker is adapted from Ref 45; apoCaM is marked with white circles and calcium binding is indicated by yellow circles. A: In the absence of calcium, apo-CaM is not associated with eNOS in which Lys497 is exposed to the trypsin digestion; the FMN domain is locked with FAD domain and electron flux is blocked. B: Upon calcium binding, wild-type CaM wraps around the eNOS CBD and potentially other sites with its two lobes bound. This binding protects Lys497 from tryptic cleavage and exposes Arg517 for trypsinolysis. Consequently, the FMN domain is released from the FAD domain, becoming accessible to the heme domain of the opposite subunit. C: With B_12Q_ mutation of N-lobe, calcium-bound C-lobe binds normally to CBD while the mutated N-lobe could not bind calcium and is unable to bring FMN domain to the heme domain of the other subunit which hinders electron transfer as illustrated by a dotted line. D: With B_34Q_ mutation of C-lobe, C-lobe is unable to bind CBD which retards electron transfer.

Taken together the results from our current study, we outlined these findings in a schematic diagram based upon NOS dimeric model from Daff [44] and CaM bi-lobed structure from Rodney *et al.* [45], illustrating how N- and C-terminal lobes of CaM differentially regulate eNOS catalysis (Figure 7). In the absence of calcium, apo-CaM is not associated with eNOS in which Lys497 is sensitive to trypsin digestion; the FMN domain, closely associated with FAD domain, is in the locked position and electron flux is blocked (Figure 7A). Upon calcium binding, wild-type CaM undergoes a conformational change; wraps around the CaM-binding domain and potentially other CaM-binding sites in eNOS with its two lobes bound, as has been proposed for the multiple CaM-binding sequences in the eNOS (15). The binding protects Lys497 from tryptic cleavage and exposes Arg517 for trypsinolysis. Consequently, the FMN domain is released from the closed to the open position, becoming accessible to the heme domain of the opposite subunit or to an exogenous electron acceptor like *cytochrome c* (Figure 7B). With B_12Q_ double mutation of N-lobe, CaM is tethered to the N-terminus of eNOS CaM-binding domain solely by calcium- bound C-lobe. This association protects Lys497 from tryptic cleavage with slight induction of the FMN domain mobility. In this state, the FMN domain is mainly in the locked position, resulting in great attenuation of eNOS activity. (Figure 7C). With B_34Q_ double mutation of C-lobe, calcium-bound N-lobe is unable to protect Lys497 from tryptic cleavage but exposes Arg517 for trypsinolysis and increases flavin fluorescence to the same level as wild-type CaM. This implies that B_34Q_ does not bind to CaM-binding domain but rather to a region adjacent to CaM-binding domain. In this state, although the FMN domain is in the open position, both eNOS oxygenase and reductase activities are greatly attenuated, indicating that the released FMN domain no longer associates with its redox partners as it normally would (Figure 7D). This clearly shows that binding between CaM and CaM-binding domain of eNOS is head-to-tail with the C-lobe of CaM bound to the N-portion of CaM-binding domain. Lobe-specific calcium binding is crucial for CaM to control eNOS electron transfer, and efficient activation requires both CaM N- and C-terminal lobes in its calcium-bound form to interact with CaM-binding domain as well as with other CaM-binding elements in eNOS.

Comparison of the calcium-binding site mutations on citrulline formation and *cytochrome c* reduction in eNOS vs. the other isoforms reveal significant differences. As shown in Table 3, single calcium-binding site mutation of CaM exerts effect on eNOS activation less than on nNOS or iNOS. Notably, B_1Q_ mutant reduced eNOS citrulline formation by 25% but completely abolished nNOS citrulline formation [21] and conversely did not influence iNOS citrulline formation [22]. On the other hand, B_3Q_ mutant which did not affect eNOS and caused a mild reduction of nNOS citrulline formation reduced iNOS citrulline formation by >50% while B_4Q_ which increased eNOS citrulline formation caused a profound reduction of nNOS but had little effect on iNOS citrulline formation. B_2Q_ did not significantly influence eNOS citrulline formation but exerted a great effect on nNOS and iNOS citrulline formation. B_3Q_ selectively reduced iNOS citrulline formation whereas B_4Q_ reduced nNOS citrulline formation. The effect of a single site mutation on *cytochrome c* reduction is concordant with that on citrulline formation with the exception of B_4Q_ which reduces cytocrome c reduction without a significant effect on citrulline formation. Our data reveal that lobe-specific mutation of calcium-binding sites (B_12Q_ and B_34Q_ alike) severely interferes with eNOS and nNOS activity without an effect on iNOS (23). These results provide evidence for differential effects of CaM calcium-binding status on electron flow and catalytic activity in eNOS vs. nNOS and iNOS.

**Table 3:** T**able 3.** Differential effect of mutating Ca^2+^-binding sites at CaM on enzyme activation among NOS isoforms.

	Cyto c Reduction			Citrulline formation or NO production		
	eNOS	nNOS	iNOS	eNOS	nNOS	iNOS
CaMs	(%)			(%)		
None	30±4^a^	4.2 b		2.8±0.8 a	0.47 b	
WT	100±12 a	100^b^	100^c^	100±2^a^	100^b^	100^c^
B_1Q_	63±7 a	11^b^	109^c^	75±4^a^	<2^b^	95^c^
B_2Q_	67±6 a	∼50^b^	64^c^	94±6^a^	26^b^	41^c^
B_3Q_	99±11 a	76^b^	67.7^c^	105±9^a^	82^b^	43^c^
B_4Q_	55±7 a	∼53^b^	42.8^c^	111±13 a	30^b^	90^c^
B_12Q_	34±4 a	∼6^d^	140^d^	24±6^a^	∼5^d^	75^d^
B_34Q_	38±5 a	∼12^d^	100^d^	18±5^a^	∼10^d^	115^d^
B_1234Q_	27±3 a	∼11^d^	<25^d^	2.3±0.4 a	<2^d^	<25^d^

a This manuscript: The activity was obtained in the presence of 0.5 μM CaM concentration. Under these conditions, the activities for eNOS bound to wild-type CaM were ∼14 min^−1^ min^−1^ for citrulline formation, and ∼286 min^−1^ for *cytochrome c* reduction.

b Data from Ref. 21.

c Data from Ref. 22.

d Data from Ref. 23.

B_4Q_ in comparison with wild-type CaM attenuated *cytochrome c* reduction by ∼45% while it increased citrulline formation (Figure 3B vs. 3A). This differential effect is unexpected as the crystallographic analysis of CaM bound to eNOS CaM-binding domain [14] or nNOS CaM-binding domain as well as CaM-iNOS FMN subdomain complex [42] has shown interaction of CaM with the canonical CaM-binding site of all three NOS isoforms in an anti-parallel orientation in which the B_4Q_ site (E140 of CaM) interacts with the N-terminal portion of CaM-binding domain situated in the vicinity NOS oxygenase domain. It is unclear how mutation of this site only affects reductase activity without reducing oxygenase activity. The results nevertheless support the notion that CaM activates reductase and oxygenase by distinct mechanisms [46].

A recent crystal structure of a CaM-bound iNOS FMN subdomain [42] has shown that Glu47 located at the CaM N-terminal lobe participates in a bridging interaction with the iNOS FMN domain that helps transduce the effects of bound CaM. The authors suggested that an analogous CaM-FMN subdomain bridging interaction be expected to be present in the nNOS and eNOS enzymes. Tejero *et al.* later proved that this bridging connection appeared to control nNOS FMN subdomain movement [43]. Here, we propose that this bridging interaction is primarily controlled by lobe-specific calcium binding in CaM. Our flavin fluorescence data demonstrate that B_34Q_ is able to induce eNOS fluorescence intensity to the same level as the wild-type CaM, suggesting an interaction between CaM Glu47 and eNOS FMN subdomain which is present in an output state. However, B_34Q_ hardly increases eNOS oxygenase and reductase activities, possibly due to defective binding to CaM-binding domain. These data imply that attachment of calcium-bound C-terminal lobe of CaM to eNOS CaM-binding domain triggers FMN domain to be in a more suitable orientation for interacting with its redox partners. As B_12Q_ induced a much smaller increase in flavin fluorescence than wild-type CaM, it is possible that the bridging interaction between CaM Glu47 and eNOS FMN subdomain requires CaM N-terminal lobe in its calcium-bound form. From these findings, we conclude that calcium-bound C-lobe and N-lobe of CaM interact with eNOS at different regions in a coordinated manner that is necessary for the efficient electron transfer among different redox centers in eNOS.

## Materials and Methods

### Materials

L-[2,3,4,5^−3^H] arginine (NET1123250UC) was obtained from PerkinElmer Inc. (6R)-5,6,7,8,-Tetrahydro-L-biopterin (H_4_B) was purchased from Research Biochemical International. Bradford protein dye reagent and electrophoretic chemicals were products of Bio-Rad. Restriction enzymes were from New Englands Biolabs. CaM-agarose, AG 50W-X8 (cation-exchange resin), NADPH and other reagents were obtained from Sigma. Sf21 insect cell was obtained from Clontech (ordering number 631411).

### Mutagenesis

The human CaM cDNA was generously provided by Dr. Emanuel E Strehler. A series of single-point mutants of human CaM with Glu (E) to Gln (Q) substitutions at the *z* positions for coordinating calcium in each EF-hand, were generated by site-directed mutagenesis and termed as B_1Q_ (E31Q), B_2Q_ (E67Q), B_3Q_ (E104Q) and B_4Q_ (E140Q), respectively. Multiple point mutants unable to bind calcium either at the N-terminal lobe (B_12Q_; E31Q and E67Q mutations), the C-terminal lobe (B_34Q_; E104Q and E140Q mutations) or both lobes (B_1234Q_; E31Q, E67Q, E104Q and E140Q) were subsequently prepared. The basic strategy for PCR-based mutagenesis was described previously [47]. The primers used for mutation and the residues changed for each mutant are listed in Table 1. The mutated residues glutamine (CAA or CAG) are highlighted with boldface. All primers were synthesized by MdBio Inc. (Taipei, Taiwan). Wild-type and mutant CaMs were cloned into pT7-7 vector through the NdeI and HindIII sites. The correct mutations were confirmed by Sequencing Service in the core facility of NHRI at Miaoli, Taiwan. The sequenced CaM genes were same as Genbank under accession number J04046.

### Expression and Purification of Wild-type and Mutant CaMs

BL21 (DE3) transformed with each CaM construct were grown at 37°C in LB medium supplemented with 100 μg/ml ampicillin. The cultures were induced by 0.3 mM isopropy-β-D-thiogalactopyranoside when the OD_600_ reached to 0.8– 1.2 and harvested after overnight expression at room temperature. Purification of various CaMs was carried out essentially as described previously [15], [48]. The CaM concentration was determined either by Bradford method [49] or spectrophotometry using Є_277 nm_  = 2560 M^−1^ cm^−1^.

### Expression and Purification of eNOS

Human eNOS cDNA were inserted into *Eco*RI site of pVL1392 transfer vector, which was used to generate recombinant viruses in Sf21/baculovirus system. Due to low heme biosynthetic capability of Sf21 cells, supplemental heme chloride (4μg/ml) was added into the culture medium 18 h postinfection to enrich heme content of the expressed eNOS. Cells were harvested 60 h postinfection, suspended in buffer containing 25 mM Tris-HCl, pH 7.5, 0.2 mM dithiothreitol (DTT), 0.1 mM EDTA, 0.1 mM EGTA, 1 μM pepstatin A, 1μM leupeptin, 1 μM antipain, 1 μM phenylmethylsulfonyl fluoride, and 10% glycerol, and then sonicated 20 s for three times, centrifuged twice at 30,000×*g* for 20 min at 4°C. The supernatant was loaded onto a 2′, 5′-ADP-Sepharose affinity column (1.5×5 cm), proteins were eluted with a buffer containing 25 mM Tris-HCl, pH 7.5, 0.1 mM DTT, 20 mM 2′&3′-AMP mixture, 0.5 M NaCl and 10% glycerol. The eluate was diluted with 4-volume buffer containing 25 mM Tris-HCl, pH 7.5, 0.1 mM DTT, 100 μM CaCl_2_ and 10% glycerol, and applied to a CaM-agarose column(1.5×5 cm) pre-equilibrated with buffer containing 25 mM Tris-HCl, pH 7.5, 0.1 mM DTT, 100 μM CaCl_2_, 100 mM NaCl and 10% glycerol (Buffer A). The column was washed with Buffer A and eluted with buffer containing 25 mM Tris-HCl, pH 7.5, 0.1 mM DTT, 2 mM EGTA, 0.5M NaCl and 10% glycerol. The eluate was concentrated by Centriprep-50 (Millipore) and dialyzed against buffer containing 25 mM Tris-HCl, pH 7.5, 0.1 mM DTT, 100 mM NaCl and 10% glycerol.

### Steady-state eNOS Activity

eNOS activity was determined by measuring conversion of L-[3H] arginine to L-[3H] citrulline as previously described [4] with slight modifications. The reaction mixture containing 25 mM Tris-HCl, pH 7.5, 0.1 mM DTT, 100 nM eNOS, 300 μM CaCl_2_, 0.2 mM EDTA, 100 μM β-NADPH, 10 μM H_4_B, 20 μM L-arginine, 10% glycerol, and 1μCi of L-[^3^H] arginine was incubated at 37°C for 4 min either with a fixed amount (0.5 μM) or various concentrations of each CaM construct. *Cytochrome c* reduction was determined at room temperature in a reaction mixture containing 25 mM Tris-HCl, pH 7.5, 100 mM NaCl, 10% glycerol, 50 μM *cytochrome c*, 0.5 μM CaM, 100 μM CaCl_2_, and 100 μM β-NADPH. The reaction was initiated by addition of 50 nM eNOS and monitored at 550 nm in a GE geneQuant100 spectrophotometer. Activity was determined using a Cred-oxi of 21 mM^−1^cm^−1^.

### Gel mobility-shift assay

A canonical CaM-binding peptide (CBD), TRKKTFKEVANAVKISASLM (eNOS residues 491–510) was synthesized by Peptide 2, Inc. (Chantilly, VA). Each CaM construct (200 pmol) was incubated with CBD at increasing molar ratios for 1 h at room temperature in a buffer (10 μl) containing 20 mM Tris, pH 7.5 with either 100 μM CaCl_2_ or 1mM EGTA. The sample was subjected to 18% nondenaturing, non-reducing PAGE [50], which was run at 30 mA under high calcium conditions (100 µM free calcium in gel buffers) or low calcium conditions (1mM EGTA in gel buffers). Binding of CBD to CaM was visualized by Coomassie blue R-250 staining.

### Fluorescence Spectroscopy

The steady-state flavin fluorescence was measured in a solution containing 25 mM Tris, pH 7.5, 100 mM NaCl, 0.1 mM DTT, 2 mM EGTA, 10% glycerol, and ∼6 μM eNOS in the absence or presence of ∼12 μM of CaM constructs using a VARIAN CARY Eclipse fluorescence spectrophotometer. A 1-ml quartz cuvette with a path length of 1 cm was used for the experiments. The total volume change was kept less than 0.3%, and the samples were maintained at room temperature during measurement. The effect of wild-type and mutant CaM binding on the eNOS flavin fluorescence was monitored upon addition of 2.5 mM CaCl_2_. The protein was excited at 455 nm and emission spectra were recorded between 460 to 700 nm within 5 min for each sample. Emission spectra were corrected for background due to the buffer and CaM effects.

### Trypsinolysis of eNOS

Limited proteolysis was carried out in a mixture containing 5 μM of recombinant eNOS purified from Sf21 cells, 50 mM Tris, pH 7.5, 100 mM NaCl, 10 μM of wild-type or mutant CaM, and either in the presence of 100 μM CaCl_2_ or 1mM EGTA. Samples were preincubated at room temperature for 15 min, and proteolysis was initiated by addition of 0.1 ng of Trypsin Gold (Promega) per pmol of eNOS. Following incubation at room temperature for 15 min, the reaction was terminated by boiling with an equal volume of 2X SDS gel-loading buffer. Proteolytic products were resolved on an 8–16% gradient SDS-PAGE [50] and visualized by staining with Coomassie Blue R250.

### N-terminal sequence analysis

Following SDS-PAGE, the tryptic samples were transferred to polyvinylidene difluoride (PVDF) membranes using CAPS buffer for 3 h at 50 V and 4°C. The bands were stained with Ponceau red and excised for sequencing. Sequencing was performed on an Applied Biosystems Model 494 protein sequencer at Mission Biotech Inc. (Taipei, Taiwan).
